# Assessment of Fasudil on Contrast-Associated Acute Kidney Injury Using Multiparametric Renal MRI

**DOI:** 10.3389/fphar.2022.905547

**Published:** 2022-06-15

**Authors:** Bin Wang, Yongfang Wang, Yan Tan, Jinxia Guo, Haoyuan Chen, Pu-Yeh Wu, Xiaochun Wang, Hui Zhang

**Affiliations:** ^1^ Department of Medical Imaging, First Hospital of Shanxi Medical University, Taiyuan, China; ^2^ Department of Medical Imaging, Shanxi Medical University, Taiyuan, China; ^3^ GE Healthcare MR Research China, Beijing, China

**Keywords:** CA-AKI, fasudil, multiparametric MRI, Rho/ROCK, renal hypoxia, renal fibrosis

## Abstract

**Aims:** To evaluate the utility of fasudil in a rat model of contrast-associated acute kidney injury (CA-AKI) and explore its underlying mechanism through multiparametric renal magnetic resonance imaging (mpMRI).

**Methods:** Experimental rats (*n* = 72) were grouped as follows: controls (*n* = 24), CA-AKI (*n* = 24), or CA-AKI + Fasudil (*n* = 24). All animals underwent two mpMRI studies (arterial spin labeling, T1 and T2 mapping) at baseline and post iopromide/fasudil injection (Days 1, 3, 7, and 13 respectively). Relative change in renal blood flow (ΔRBF), T1 (ΔT1) and T2 (ΔT2) values were assessed at specified time points. Serum levels of cystatin C (CysC) and interleukin-1β (IL-1β), and urinary neutrophil gelatinase-associated lipocalin (NGAL) concentrations were tested as laboratory biomarkers, in addition to examining renal histology and expression levels of various proteins (Rho-kinase [ROCK], α-smooth muscle actin [α-SMA]), hypoxia-inducible factor-1α (HIF-1α), and transforming growth factor-β1 (TGF-β1) that regulate renal fibrosis and hypoxia.

**Results:** Compared with the control group, serum levels of CysC and IL-1β, and urinary NGAL concentrations were clearly increased from Day 1 to Day 13 in the CA-AKI group (all *p* < 0.05). There were significant reductions in ΔT2 values on Days 1 and 3, and ΔT1 reductions were significantly more pronounced at all time points (Days 1–13) in the CA-AKI + Fasudil group (vs. CA-AKI) (all *p* < 0.05). Fasudil treatment lowered expression levels of ROCK-1, and p-MYPT1/MYPT1 proteins induced by iopromide, decreasing TGF-β1 expression and suppressing both extracellular matrix accumulation and α-SMA expression relative to untreated status (all *p* < 0.05). Fasudil also enhanced PHD2 transcription and inhibition of HIF-1α expression after CA-AKI.

**Conclusions:** In the context of CA-AKI, fasudil appears to reduce renal hypoxia, fibrosis, and dysfunction by activating (Rho/ROCK) or inhibiting (TGF-β1, HIF-1α) certain signaling pathways and reducing α-SMA expression. Multiparametric MRI may be a viable noninvasive tool for monitoring CA-AKI pathophysiology during fasudil therapy.

## Introduction

Intravenous iodinated contrast media (CM) are used ubiquitously in computed tomography (CT) and angiographic studies as a means of evaluating disease progression or determining treatment response. Despite their benefits, there is a possibility of contrast-associated acute kidney injury (CA-AKI) and subsequent long-term renal damage (Davenport et al., 2020). According to the Kidney Disease Improving Global Outcomes (KDIGO) Clinical Practice Guidelines, CA-AKI is defined as a rise (relative to baseline) in serum creatinine (Cr) after CM exposure, marked by a minimum increase of 0.3 mg/dL (or 26.5 µmol/L) within 2 days or a greater than 1.5-fold increase within 7 days (Kusirisin et al., 2020). The incidence of CA-AKI reportedly ranges from 1 to 25% in hospital-acquired cases of AKI, constituting the third most common cause of AKI leading to prolonged hospitalization of inpatients (Pattharanitima and Tasanarong 2014; Wang et al., 2017). Although numerous studies have linked CA-AKI to a significantly higher relative risk of serious, adverse, and short- or long-term outcomes (Weisbord et al., 2020), pertinent clinical practice guidelines offer no definitive treatments for established CA-AKI (Stacul et al., 2011). The need for novel therapeutic regimens is therefore urgent.

Fasudil is a derivative of 5-isoquinoline sulfonamide and an inhibitor of Rho-kinase (ROCK) that exerts a wide range of pharmacologic effects (Xia et al., 2019). Its utility in the setting of diabetic nephropathy (Xie et al., 2020), chronic kidney disease (Kushiyama et al., 2013), and drug-induced kidney injury (Park et al., 2011) has been amply documented. ROCK is a protein kinase that triggers the Rho/ROCK signaling pathway (Kusirisin et al., 2020). Results of several recent studies have indicated that Rho/ROCK signaling blockade mitigates a number of adverse events, including inflammation, hypoxia, oxidative effects, and fibrosis (Matoba et al., 2013; Wang et al., 2018). The mechanisms driving CA-AKI are not completely clear, but there is evidence that tubulointerstitial inflammation, fibrosis, and medullary hypoxia are important factors (Zhang et al., 2018; Wang et al., 2019b). This study documents the renoprotective effects of ROCK inhibition in a rat model of CA-AKI and explores mechanisms that may perpetuate these effects over relatively long periods.

Multiparametric magnetic resonance imaging (mpMRI) is a new and rapidly evolving technique capable of detecting near-real-time kidney changes and enabling quantitation of fasudil effects in conjunction with CA-AKI (Notohamiprodjo et al., 2010; Leung et al., 2017). Arterial spin labeling (ASL) utilizes blood-based water molecules as tracers to measure renal perfusion (Williams et al., 1994; Rajendran et al., 2013; Tan et al., 2015; Liang et al., 2016; Zhao et al., 2021). T1 mapping is capable of depicting even small variations of T1 relaxometry within the tissue, having been used to assess ischemia-reperfusion AKI (Hueper et al., 2014), renal fibrosis in rats with unilateral ureteral obstruction (Hu et al., 2019), and renal transplantation in both animal models and humans (Hueper et al., 2016; Peperhove et al., 2018). T1 mapping is also of reputed utility in measuring renal fibrosis, featuring the potential to differentiate the various stages of AKI (Hueper et al., 2014). T2 mapping, with a voxel-wise evaluation of proton spin-spin relaxation times, provides a noninvasive means of visualizing and quantifying tissue composition (Adams et al., 2019). Inflammation and edema may be visualized in T2-weighted MRI sequences (Hueper et al., 2013). Applications of ASL and T1/T2 mapping are important in researching renal perfusion, fibrosis, inflammation, and edema during fasudil treatment of CA-AKI.

For our purposes, we used all three methods (ASL, T1, and T2 mapping) to study the mechanisms of ROCK inhibition by fasudil in a rat model of CA-AKI. We specifically addressed certain signaling pathways (Rho/ROCK, hypoxia-inducible factor-1α [HIF-1α], and transforming growth factor-β1 [TGF-β1]), and α-smooth muscle actin (α-SMA) expression, all of which influence renal hypoxia, renal fibrosis, and renal function.

## Materials and Methods

### Animal Handling

This study was performed in compliance with the guidelines of the Institute for Laboratory Animal Research at Shanxi Medical University, and the university’s Committee on the Ethics of Animal Experiments approved our protocol. All male SD rats (8-week-old; weight range: 180–220 g; *n* = 72) were purchased from the animal laboratory center of Shanxi Medical University (SYXK (jin) 2019-0007). We housed rats in cages (*n* = 3/each) in a laboratory environment, maintaining 12-h light/dark cycles at 20 °C. Water and food were always available to the rats. Termination criteria were weight loss up to 20–25%, complete loss of appetite for 24 h or poor appetite (less than 50% of normal) for 3 days, and mental depression accompanied by hypothermia (below 37°C).

### Experimental Design

**FIGURE 1:**
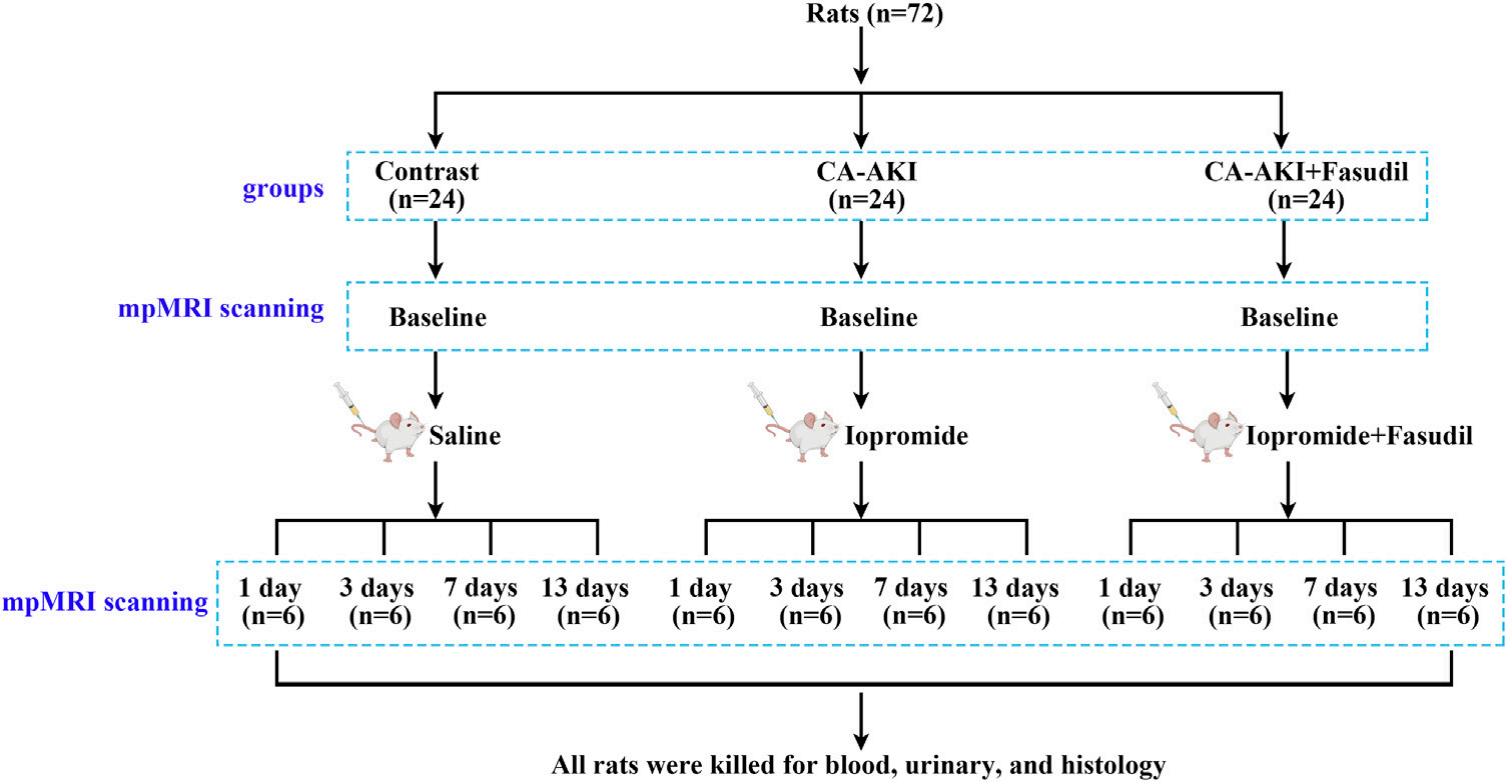
Experimental flow chart. Flow chart shows the group designations, and the time points for mpMRI data acquisition, blood, urine, and kidney tissue collections. mpMRI = multiparametric magnetic resonance imaging.

All animals (*n* = 72) were acclimatized for 7 days in advance of experimentation. The CA-AKI model was achieved as previously described through tail-vein injections of iopromide (8 g/kg organically bound iodine, 370 mg/mL; Bayer Healthcare, Berlin, Germany) (Wang et al., 2014). The rats were randomly grouped (*n* = 24 each) as controls (normal saline injection only), CA-AKI (iopromide injection), or CA-AKI + Fasudil (fasudil injection [10 mg/kg; Selleckchem, Houston, TX, United States] pre- and post-injection of iopromide) (Wang et al., 2018). The latter was further divided into four subgroups according to four time points: day 1, 3, 7, and 13 upon the administration of fasudil/iopromide (*n* = 6 each). Fasudil injections were intravenous, performed before (12 and 2 h) and after (4 h) iopromide injection (Wang et al., 2018). Anesthesia was induced by pentobarbital sodium via intraperitoneal injection (3%, 2 mL/kg). A schematic representation of our study is shown in Figure 1.

### Arterial Spin Labeling, T1/T2-Mapping, and Data Analysis

**FIGURE 2:**
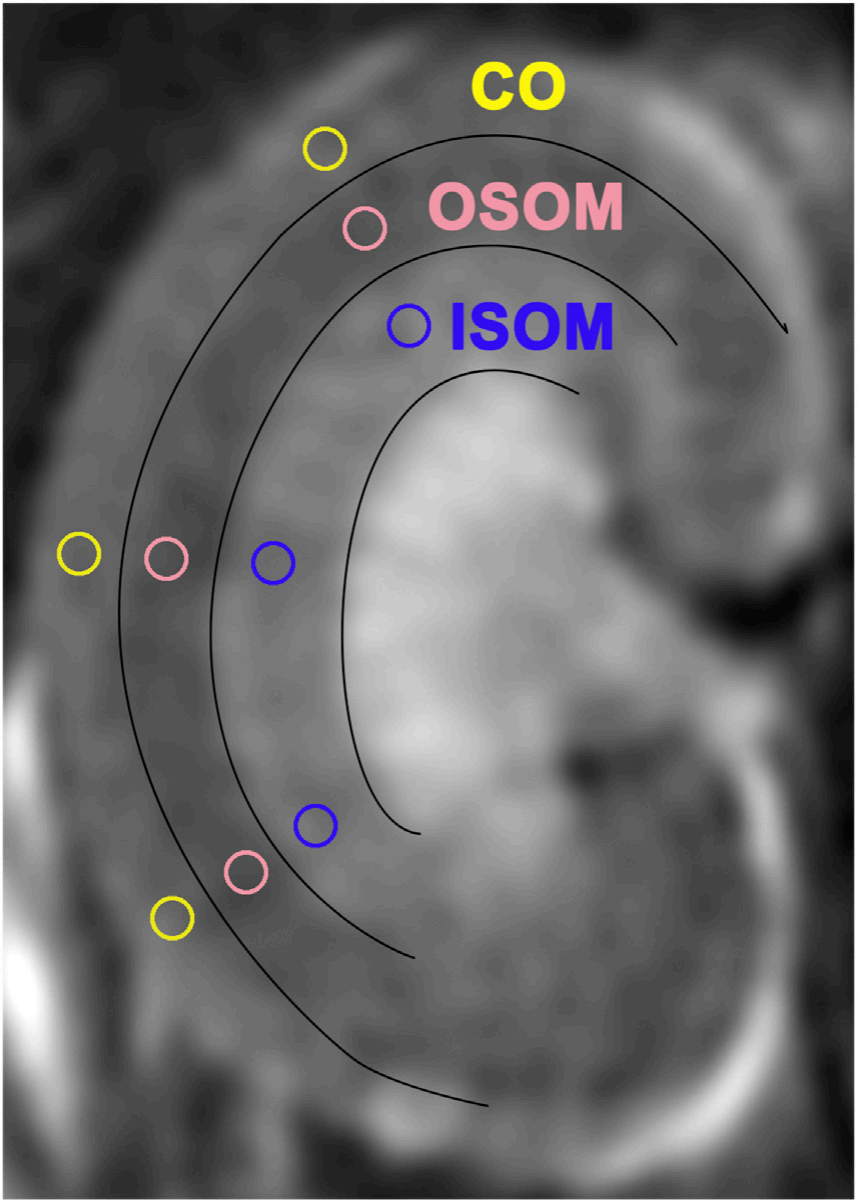
Regions of interest (ROIs) in three sectors of T2-weighted images. ISOM: inner stripe of the outer medulla; CO: cortex; OSOM: outer stripe of the outer medulla.

Multiparametric MRI studies, including ASL and T1/T2-mapping, were performed on a scanner (Discovery 750w 3.0-T; GE Healthcare, Waukesha, WI, United States) by employing a 5-cm rodent coil. The inversion recovery method was applied for T1 mapping, since it is insensitive to inaccuracies in excitation flip angles or imperfect spoiling and additional calibration measurements was usually not required (Stikov et al., 2015). A 2D FSE multi-echo sequence was used for T2 mapping and eight echoes were acquired, which referred the method in reference (Ma et al., 2019), where the T2 mapping method has been validated with the ultrasmall superparamagnetic iron oxide (USPIO) phantom. Parameters of data acquisition through ASL and T1/T2 mapping are shown in Table 1. In each subgroup, rats underwent mpMRI at baseline (before iopromide/fasudil injection) and variably on Days 1, 3, 7, or 13 after iopromide/fasudil injection. During the MRI procedure, the rats were placed supine inside the coil. The body temperature was continuously monitored and maintained at 37.0 ± 0.5°C using a heating lamp (Anhongda Optoelectronic Technology Co. Ltd., Shenzhen, China). Heart rate, blood oxygen, respiration rate, and blood pressure were noninvasively monitored by an animal multifunctional equipment (Yuyan Instruments, Shanghai, China). The T1 and T2 relaxometry were calculated by respectively fitting the multi-inverse time T1 mapping and multi-echo T2 mapping data with home-written Matlab code (version 2014b). The method for T1 relaxometry is the reduced-dimension non-linear least squares (RD-NLS) algorithm proposed by Barral et al. (Barral et al., 2010) and modified based on the code of qMRLab (https://qmrlab.org/) while it for T2 relaxometry is the least-squares fitting by using equation S=S_0_*exp (-TE/T2) (Dou et al., 2018). The renal blood flow (RBF) was generated automatically with vendor-provided algorithm (Artz et al., 2011; Kim et al., 2017) in the scanning console. Quantitative regional RBF and T1/T2 values in region-of-interest (ROI) were measured manually by two seasoned radiologists blinded to group assignments. On the central coronal slice of right kidney, three ROI markers were respectively placed on the upper, middle, and inferior renal regions within the cortex (CO), inner stripe of the outer medulla (ISOM), and the outer stripe of the outer medulla (OSOM) with the same shape and size (approx. 1.5–2.0 mm^2^) using a published method (Wang et al., 2022) (Figure 2). Adjusted ΔRBF, ΔT1, and ΔT2 were calculated by subtracting baseline RBF, T1, and T2 values from post-injection RBF, T1, and T2 values at specified time points [for example: ΔRBF = RBF (post-injection)—RBF (baseline)].

**TABLE 1:** Scanning parameters for mpMRI sequences.

	T1-mapping	T2-mapping	ASL
Sequence type	IR-FSE	T2map	FAIR SE-EPI
Orientation	Coronal	Coronal	Axial
Repetition time (TR), ms	2000	600	1,300
Echo time (TE), ms	8.5	7.89, 15.78, 23.68, 31.57, 39.46, 47.35, 55.24, 63.14	30.4
Inversion time (TI), ms	50, 100, 200, 500, 800, 1,100, 1,500, 1800	---	700 ms
No. of slices	2	4	2
Bandwidth, hertz per pixel	35.71	62.5	125.0
Echo train length	3	1	---
Field of view, cm^2^	8*6.4	10*10	10*8
Matrix	192*192	256*256	128*100
Section thickness, mm	2	2	5
Slice spacing, mm	0.5	0.5	0.5
Number of excitations	2	2	20
Acquisition time	3 min 32 s/TI	5 min10 s	55 s

mpMRI, multiparametric magnetic resonance imaging; SE-EPI, spin-echo echo-planar imaging; FAIR, flow-sensitive alternating inversion recovery; IR-FSE, inversion-recovery fast spin-echo; TI, inversion time.

### Laboratory Measurements

Following MRI scans, we collected blood from the inferior vena cava and urine from the bladder. These samples were stored at −80 °C. Serum levels of cystatin C (CysC) and interleukin-1β (IL-1β), and urinary levels of neutrophil gelatinase-associated lipocalin (NGAL) were quantified using enzyme-linked immunosorbent assay (ELISA) kits (CysC, MM-0438R1; IL-1β, MM-0047R1; NGAL, MM-0271R1; Meimian Industrial Co. Ltd., Jiangsu, China) following manufacturer protocols.

### Histologic Preparations

On Days 1, 3, 7, and 13, we sacrificed six rats by injecting of anesthesia overdose per subgroup for histologic examination. The kidneys on the right side were resected and fixed in paraformaldehyde (4%) for 3 days, then embedded in paraffin, sectioned, and stained [Masson’s trichrome and hematoxylin/eosin (H&E)] for light microscopy. As a semiquantitative gauge of kidney changes, five high-power (400×) fields of the renal cortex (CO) and outer/inner stripe of the outer medulla (OSOM and ISOM) were inspected. Tissue injury was scored (as previously described) on the following levels: 0, none; 1, mild (<25%); 2, moderate (<50%); 3, severe (<75%); or 4, very severe (>75%) (Ulusoy et al., 2014). Interstitial fibrosis was also assessed in five random, non-overlapping fields stained with Masson’s trichrome, using ImageJ software (National Institutes of Health, Bethesda, MD, United States) to decide the relative percentage contributed by collagenized (blue-stained) tissue.

### Immunologic Changes

Open deparaffinization and rehydration, the sections were first treated in 3% hydrogen peroxide to inactivate endogenous peroxidase. After antigen retrieval via boiling in sodium citrate buffer (pH = 6.0) or EDTA, the sections were soaked with normal goat serum (5%), and subsequently incubated with specific antibodies: ROCK-1 (1:100, sc-17794; Santa Cruz Biotechnology), α-SMA (1:500, 19,245; Cell Signaling Technology), and HIF-1α (1:200, ab2185; Abcam). ROCK-1, α-SMA, and HIF-1α expression levels were quantified (%) under ×400 magnification using ImageJ software.

### Western Blot

Ultimately, we selected the 3-day subgroup to investigate the mechanisms of the fasudil effect in our rat model of CA-AKI. All left kidneys of the three animal groups were collected on Day 3, and the protein concentrations were determined by the bicinchoninic acid method. We then used equal aliquots (∼10 µg) of boiled protein samples for electrophoretic separation (8–12% sodium dodecyl sulfate (SDS) polyacrylamide gel) and western blot. The primary antibodies were: ROCK-1 (1:500, sc-17794; Santa Cruz Biotechnology); ROCK-2 (1:1,000, abs136978; Absin Bioscience); phosphorylated myosin light chain phosphatase (p-MYPT1: 1:1,000, 4,563; Cell Signaling Technology); myosin light chain phosphatase (MYPT1: 1:1,000, abs131546; Absin Bioscience); prolyl-4-hydroxylase domain-containing protein 2 (PHD2: 1:1,000, abs151871; Absin Bioscience); HIF-1α (1:1,000, ab2185; Abcam); TGF-β1 (1:1,000, ab215715; Abcam); Smad3 (1:1,000, 9,523; Cell Signaling Technology); p-Smad3 (1:1,000, 9,520; Cell Signaling Technology); and α-SMA (1:1,000, 19,245; Cell Signaling Technology), followed by peroxidase-labeled conjugated secondary mouse (1:10,000, RS0001; ImmunoWay Biotechnology) or rabbit (1:10,000, RS0002; ImmunoWay Biotechnology) antibodies for 2 h. Specific protein bands were scanned in a western blotting detection device. Band areas were analyzed with ImageJ. The internal control was beta-actin.

### Data Analysis

All computations were carried out using SPSS v22.0 software (IBM Corp, Armonk, NY, United States). Measured data were expressed as mean ± standard deviation values. To assess between-group differences in numerical data, the one-way analysis of variance was applied, and the Bonferroni or Tamhane’s T2 post hoc tests were performed for further multiple pairwise comparisons. Mann-Whitney and Kruskal-Wallis tests were used to analyze the unpaired data. Spearman’s correlation analysis was used to assess the relationship between mpMRI parameters and histological changes and HIF-1α expression. *p* < 0.05 was defined as significance.

## Results

Except for 2 rats died 7 days post intravascular injection of iopromide (CA-AKI group), and 1 rat died 13 days post intravascular injection of saline (Control group), a total of 72 rats were completed in the study at last. All the data were successfully recorded. In the data analysis, we set the baseline values of ΔT1, ΔT2, and ΔRBF to zero to exclude the effects of variations from the baseline.

### Effects of Fasudil on RBF and T1/T2 Relaxation Times

**FIGURE 3:**
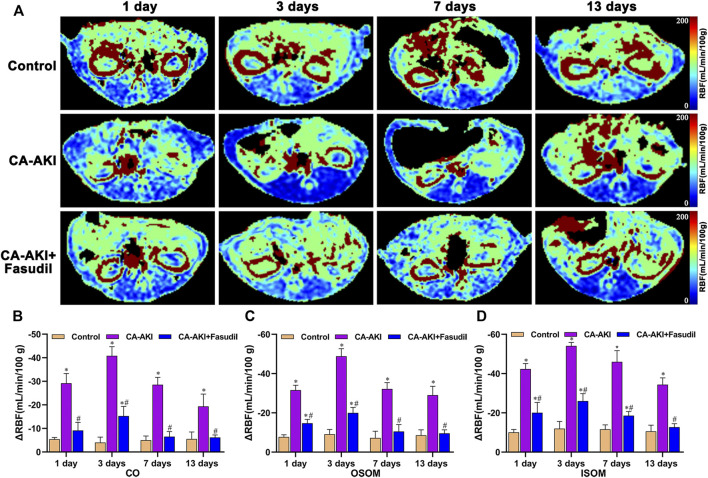
Representative ASL maps in three animal groups: **(A)** Parametric images obtained from ASL maps and **(B–D)** quantitative analysis of ΔRBF values at CO, OSOM, and ISOM. The same window level, and width are displayed for ASL maps. Signal changes are scaled from 0 (blue) to 200 (red), representing a range of RBF values. ^*^ *p* < 0.05 between CA-AKI/CA-AKI + Fasudil and controls; ^#^ *p* < 0.05 between CA-AKI + Fasudil and CA-AKI. ASL: arterial spin labeling; CA-AKI: contrast-associated acute kidney injury; RBF: renal blood flow; ISOM: inner stripe of the outer medulla; CO: cortex; OSOM: outer stripe of the outer medulla.

**FIGURE 4:**
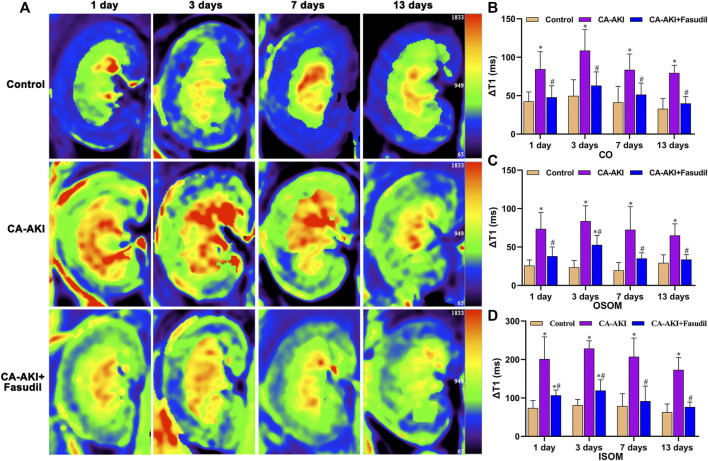
Representative T1 mapping outcomes in three animal groups: **(A)** Parametric images obtained by T1 mapping and **(B–D)** quantitative analysis of ΔT1 values at CO, OSOM, and ISOM.^*^ *p* < 0.05 between CA-AKI/CA-AKI + Fasudil and controls; ^#^ *p* < 0.05 between CA-AKI + Fasudil and CA-AKI. CA-AKI: contrast-associated acute kidney injury; ISOM: inner stripe of the outer medulla; CO: cortex; OSOM: outer stripe of the outer medulla.

**FIGURE 5:**
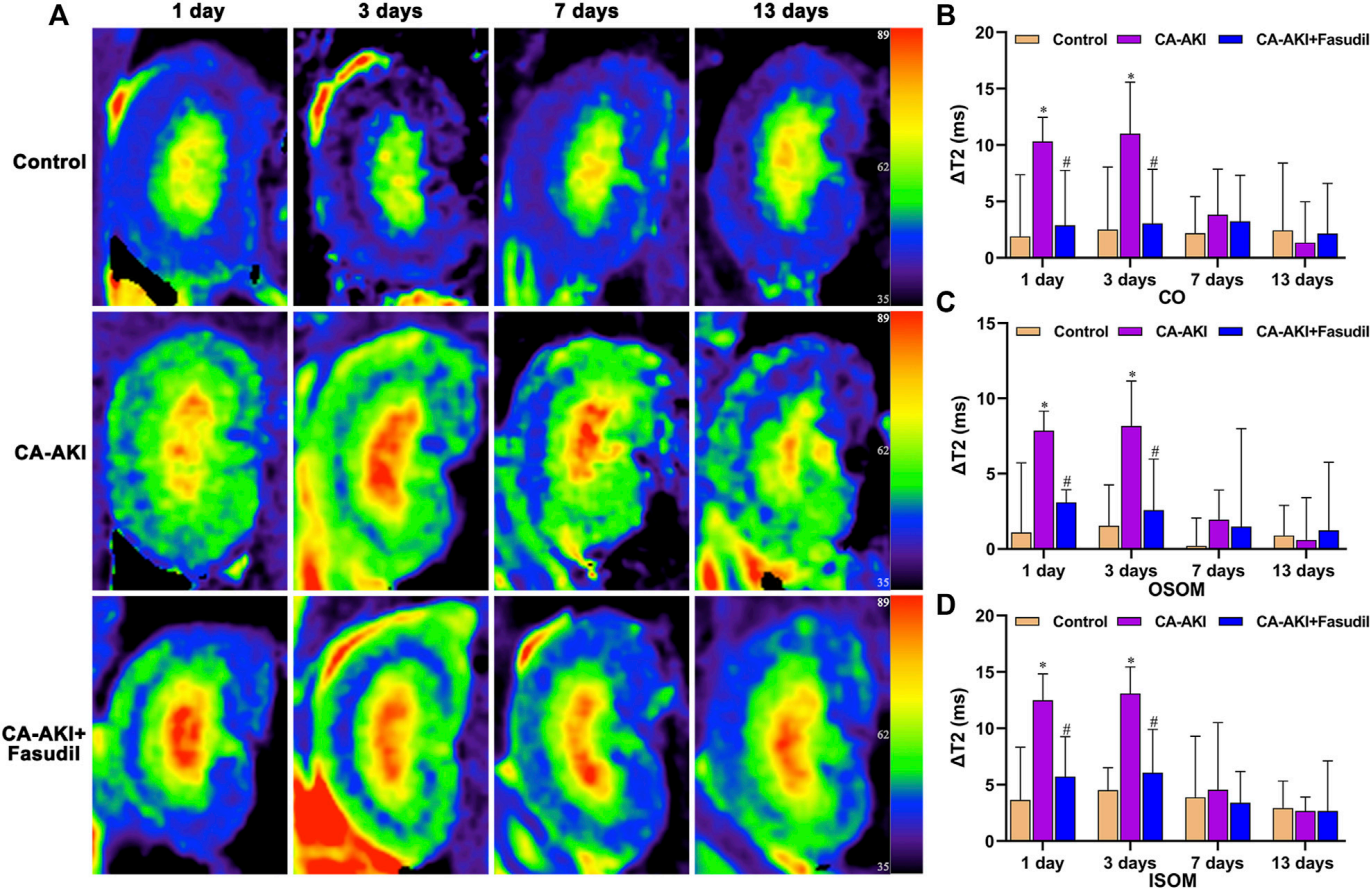
Representative T2 mapping outcomes in three animal groups: **(A)** Parametric images obtained by T2 mapping and **(B–D)** quantitative analysis of ΔT2 values at CO, OSOM, and ISOM.^*^ *p* < 0.05 between CA-AKI/CA-AKI + Fasudil and controls; ^#^ *p* < 0.05 between CA-AKI + Fasudil and CA-AKI. CA-AKI: contrast-associated acute kidney injury; ISOM: inner stripe of the outer medulla; CO: cortex; OSOM: outer stripe of the outer medulla.

In the study, significant differences were observed in comparisons of baseline renal blood flow (RBF), T1, and T2 values (Supplementary Table S1). ΔRBF, ΔT1, and ΔT2 values for CO, OSOM, and ISOM at the post-injection time points are summarized by the group in Table 2 and illustrated in Figures 3–5. There was an upward trend for ΔT1 and ΔT2 in the early phase (Days 1 and 3) that diminished as CA-AKI developed. An initial drop in ΔRBF (Day 1) subsequently reversed from Day 3 onward at all three renal zones.

**TABLE 2:** Overview of 13-day multiparametric zonal changes in kidneys for three animal groups.

		1 day (*n* = 6)	3 days (*n* = 6)	7 days (*n* = 6)	13 days (*n* = 6)
**ΔT1 (ms)**					
**CO**	Control	42.546 ± 12.210	49.757 ± 20.981	41.330 ± 20.887	32.934 ± 13.330
	CA-AKI	84.520 ± 22.642^*^	108.727 ± 27.360^*^	83.698 ± 20.483^*^	79.579 ± 9.930^*^
	CA-AKI + Fasudil	47.950 ± 14.811^#^	63.206 ± 17.713^#^	51.378 ± 14.904^#^	39.880 ± 8.923^#^
**OSOM**	Control	25.943 ± 7.231	23.604 ± 8.985	19.686 ± 10.252	29.492 ± 10.311
	CA-AKI	73.710 ± 21.372^*^	83.717 ± 20.015^*^	72.566 ± 29.986^*^	65.124 ± 15.117^*^
	CA-AKI + Fasudil	38.215 ± 11.874^#^	52.750 ± 12.404^*#^	35.239 ± 7.347^#^	33.784 ± 6.520^#^
**ISOM**	Control	73.819 ± 19.631	81.200 ± 15.095	78.830 ± 32.246	62.808 ± 21.624
	CA-AKI	201.366 ± 57.468^*^	228.717 ± 20.220^*^	207.484 ± 48.341^*^	173.197 ± 32.251^*^
	CA-AKI + Fasudil	106.775 ± 13.898^*#^	119.274 ± 28.220^*#^	91.823 ± 39.066^#^	76.661 ± 12.708^#^
**ΔT2 (ms)**					
**CO**	Control	1.901 ± 5.471	2.514 ± 5.540	2.197 ± 3.227	2.439 ± 5.974
	CA-AKI	10.328 ± 2.123^*^	11.007 ± 4.566^*^	3.832 ± 4.043	1.366 ± 3.606
	CA-AKI + Fasudil	2.889 ± 4.875^#^	3.059 ± 4.792^#^	3.243 ± 4.081	2.164 ± 4.449
**OSOM**	Control	1.127 ± 4.125	1.695 ± 5.921	0.208 ± 1.847	0.889 ± 2.003
	CA-AKI	7.792 ± 0.538^*^	8.786 ± 4.180^*^	1.949 ± 1.972	0.594 ± 2.881
	CA-AKI + Fasudil	2.060 ± 4.470^#^	2.576 ± 3.392^#^	1.477 ± 6.571	1.229 ± 4.531
**ISOM**	Control	3.657 ± 4.667	4.530 ± 1.979	3.898 ± 5.410	2.945 ± 2.383
	CA-AKI	12.495 ± 2.338^*^	13.093 ± 2.349^*^	4.563 ± 5.943	2.677 ± 1.236
	CA-AKI + Fasudil	5.718 ± 3.536^#^	6.077 ± 3.839^#^	3.402 ± 2.788	2.670 ± 4.450
**ΔRBF (mL/min/100g)**					
**CO**	Control	−0.545 ± 0.683	−4.022 ± 2.305	−5.037 ± 1.732	−5.529 ± 2.943
	CA-AKI	−29.192 ± 4.110^*^	−40.831 ± 3.860^*^	−28.565 ± 3.110^*^	−19.358 ± 5.184^*^
	CA-AKI + Fasudil	−9.156 ± 3.386^#^	−15.276 ± 4.039^*#^	−6.541 ± 2.121^#^	−6.204 ± 1.037^#^
**OSOM**	Control	−7.747 ± 1.065	−9.139 ± 2.371	−7.212 ± 3.416	−8.565 ± 2.745
	CA-AKI	−31.560 ± 2.508^*^	−48.820 ± 3.846^*^	−32.190 ± 3.173^*^	−29.018 ± 4.508^*^
	CA-AKI + Fasudil	−14.721 ± 1.936^*#^	−19.960 ± 2.827^*#^	−10.537 ± 3.465^#^	−9.573 ± 1.753^#^
**ISOM**	Control	−10.096 ± 1.446	−11.955 ± 3.668	−11.606 ± 2.275	−10.597 ± 3.149
	CA-AKI	−42.337 ± 2.796^*^	−54.051 ± 1.834^*^	−45.966 ± 5.729^*^	−34.394 ± 3.434^*^
	CA-AKI + Fasudil	−20.073 ± 5.212^*#^	−26.038 ± 3.726^*#^	−18.587 ± 2.079^*#^	−12.705 ± 1.746^#^

*p< 0.05 between CA-AKI/CA-AKI + Fasudil and controls.

^#^p < 0.05 between CA-AKI + Fasudil and CA-AKI.

Mean ± SD of ΔT1 values, ΔT2 values, and ΔRBF values in cortex.

CO, cortex; OSOM, outer stripe of the outer medulla; ISOM, inner stripe of the outer medulla.

As shown in Figure 3, renal parenchymal ΔRBF values were significantly decreased at all time points in the CA-AKI group, compared with healthy controls or with the CA-AKI + Fasudil group. On Day 1 after iopromide injection, the decrease in RBF in the CA-AKI group is significantly larger than in controls or in the CA-AKI + Fasudil group (all *p* < 0.0001). By Day 13, ΔRBF values still differed significantly for ISOM, OSOM, and CO between CA-AKI and CA-AKI + Fasudil groups (all *p* < 0.0001).

In terms of ΔT1 values, significant differences observed initially (Day 1) in the CA-AKI group persisted thereafter (Days 3–13). Compared with the CA-AKI group, fasudil-treated rats displayed significant less increases in T1 throughout the duration (Days 1–13). Values of ΔT1 for CO (*p* = 0.006), OSOM (*p* = 0.002), and ISOM (*p* = 0.027) were significantly lower in the CA-AKI + Fasudil (vs. CA-AKI) group on Day 1 and remained significantly lower (CO, *p* = 0.009; OSOM, *p* = 0.007; ISOM, *p* < 0.0001) on Day 3. By Day 13, the between-group difference in ΔT1 had broadened considerably for ISOM, OSOM, and CO (all *p* ≤ 0.001) (Figure 4).

On Days 1 and 3, ΔT2 values for CO, OSOM, and ISOM were significantly increased in the CA-AKI (vs. control) group (all *p* < 0.05) but showed no significant zonal increases on Days 7 and 13 (all *p* > 0.05). In the CA-AKI + Fasudil (vs. CA-AKI) group, zonal ΔT2 values exhibited significant declines on Day 1 (CO, *p* = 0.031; OSOM, *p* < 0.0001; ISOM, *p* = 0.017) and on Day 3 (CO, *p* = 0.044; OSOM, *p* = 0.018; ISOM, *p* = 0.002); there were no significant differences in ΔT2 values of the two groups on Day 7 or 13 (all *p* > 0.05) (Figure 5).

### Effects of Fasudil on Kidney Function

As the three excellent-performing core markers of CA-AKI, serum levels of CysC and IL-1β, and urinary NGAL concentration significantly increased for the duration (Days 1–13) of testing (Table 3). Significant differences in IL-1β levels were evident among the three groups. By Day 13, none of the untreated rats had completely normalized to baseline, compared with controls. Serum concentrations of CysC were significantly higher in the CA-AKI (vs. controls) group on Days 1–7 (Day 1, *p* < 0.0001; Day 3, *p* < 0.0001; Day 7, *p* < 0.0001), and significance were sustained on Day 13 (*p* = 0.002). However, there were no significant difference in CA-AKI + Fasudil (vs. CA-AKI) group on Days 1–13 (all *p* > 0.05). Urinary NGAL showed a similar pattern. Rats injected iopromide displayed higher levels of urinary NGAL, compared with the control group, reaching significance at each time point (Day 1, *p* < 0.0001; Day 3, *p* < 0.0001; Day 7, *p* < 0.0001; Day 13, *p* = 0.001); while, NGAL was significantly lower in the CA-AKI + Fasudil (vs. CA-AKI) group only on Day 13 (*p* = 0.011). Serum IL-1β levels were significantly lower in the CA-AKI + Fasudil (vs. CA-AKI) group at all time points (Day 1, *p* < 0.0001; Day 3, *p* < 0.0001; Day 7, *p <* 0.0001; Day 13, *p* = 0.019). The CA-AKI + Fasudil group did not differ significantly from controls (*p* = 0.139) by Day 13 (Table 3).

**TABLE 3:** Laboratory data for three animal groups.

		1 day (*n* = 6)	3 days (*n* = 6)	7 days (*n* = 6)	13 days (*n* = 6)
**CysC (ng/ml)**	Control	407.87 ± 37.67	412.88 ± 50.08	413.58 ± 36.76	408.10 ± 35.98
	CA-AKI	609.96 ± 27.82^*^	581.86 ± 36.18^*^	552.63 ± 31.36^*^	482.21 ± 28.25^*^
	CA-AKI + Fasudil	568.03 ± 33.29^*^	528.91 ± 33.31^*^	509.31 ± 27.94^*^	438.97 ± 26.10
**NGAL (ng/ml)**	Control	17.82 ± 2.48	18.18 ± 2.87	17.57 ± 2.16	17.12 ± 1.87
	CA-AKI	31.81 ± 3.02^*^	28.84 ± 2.84^*^	26.92 ± 2.32^*^	23.65 ± 2.85^*^
	CA-AKI + Fasudil	27.78 ± 2.85^*^	24.77 ± 2.91^*^	23.43 ± 2.49^*^	19.84 ± 2.03^#^
**IL-1β (ng/ml)**	Control	55.88 ± 5.92	52.17 ± 6.78	55.26 ± 3.91	52.20 ± 4.94
	CA-AKI	126.55 ± 6.23^*^	116.71 ± 6.11^*^	91.12 ± 3.79^*^	81.12 ± 13.17^*^
	CA-AKI + Fasudil	95.65 ± 6.93^*#^	84.16 ± 7.30^*#^	64.13 ± 5.03^*#^	57.85 ± 3.58^#^

*p< 0.05 between CA-AKI/CA-AKI + Fasudil and controls.

^#^p < 0.05 between CA-AKI + Fasudil and CA-AKI.

Mean ± SD of serum CysC, IL-1β, and urinary NGAL.

### Effects of Fasudil on Renal Histopathology

Unlike controls, HE-stained kidney sections from rats with CA-AKI revealed pronounced tubular vacuolization, inflammatory cell infiltrates, exfoliated renal tubular epithelial cells, tubular cell necrosis, tubular luminal cast formation, glomerular atrophy, and renal interstitial fibrosis, all expected changes in this context. However, the pathology was significantly less severe in rats given fasudil during the same period.

**FIGURE 6:**
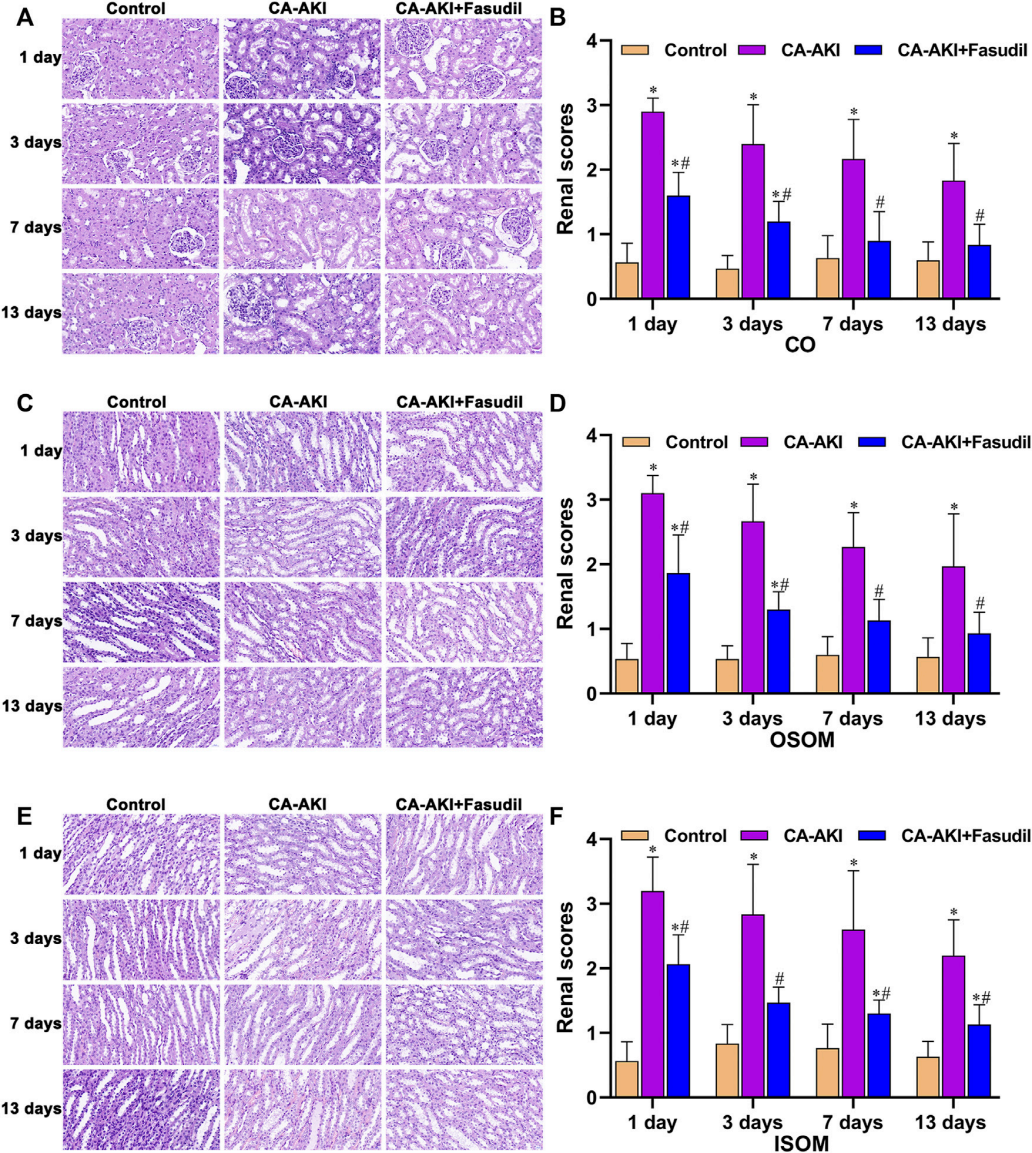
Renal histopathology in three animal groups: **(A)** microscopic views of glomerular and tubular injury at CO; **(B)** histologic scores at CO; **(C)** microscopic views of tubular injury at OSOM; **(D)** histologic scores at OSOM; **(E)** microscopic views of tubular injury at ISOM; and **(F)** histologic scores at ISOM.^*^ *p* < 0.05 between CA-AKI/CA-AKI + Fasudil and controls; ^#^ *p* < 0.05 between CA-AKI + Fasudil and CA-AKI. CA-AKI: contrast-associated acute kidney injury; ISOM: inner stripe of the outer medulla; CO: cortex; OSOM: outer stripe of the outer medulla.

Semiquantitative scores of histologic changes were significantly lower in fasudil-treated (vs. untreated) rats with CA-AKI for the duration (Figure 6). Scored renal injuries at CO were milder in the CA-AKI + Fasudil (vs. CA-AKI) group on Day 1 (*p* < 0.0001) and remained on Days 7 and 13 (Day 7, *p* = 0.001; Day 13, *p* = 0.002) (Figures 6A,B). At OSOM, renal injury scores differed significantly in CA-AKI + Fasudil and CA-AKI groups at all time points (Day 1, *p* < 0.0001; Day 3, *p* < 0.0001; Day 7, *p* = 0.001; Day 13, *p* = 0.013) (Figures 6C,D). At ISOM, a pronounced reduction in renal injury scores observed for the CA-AKI + Fasudil (vs. CA-AKI) group on Days 1 and 3 (both *p* = 0.001) was sustained until Day 13 (*p* = 0.01) (Figures 6E,F).

**FIGURE 7:**
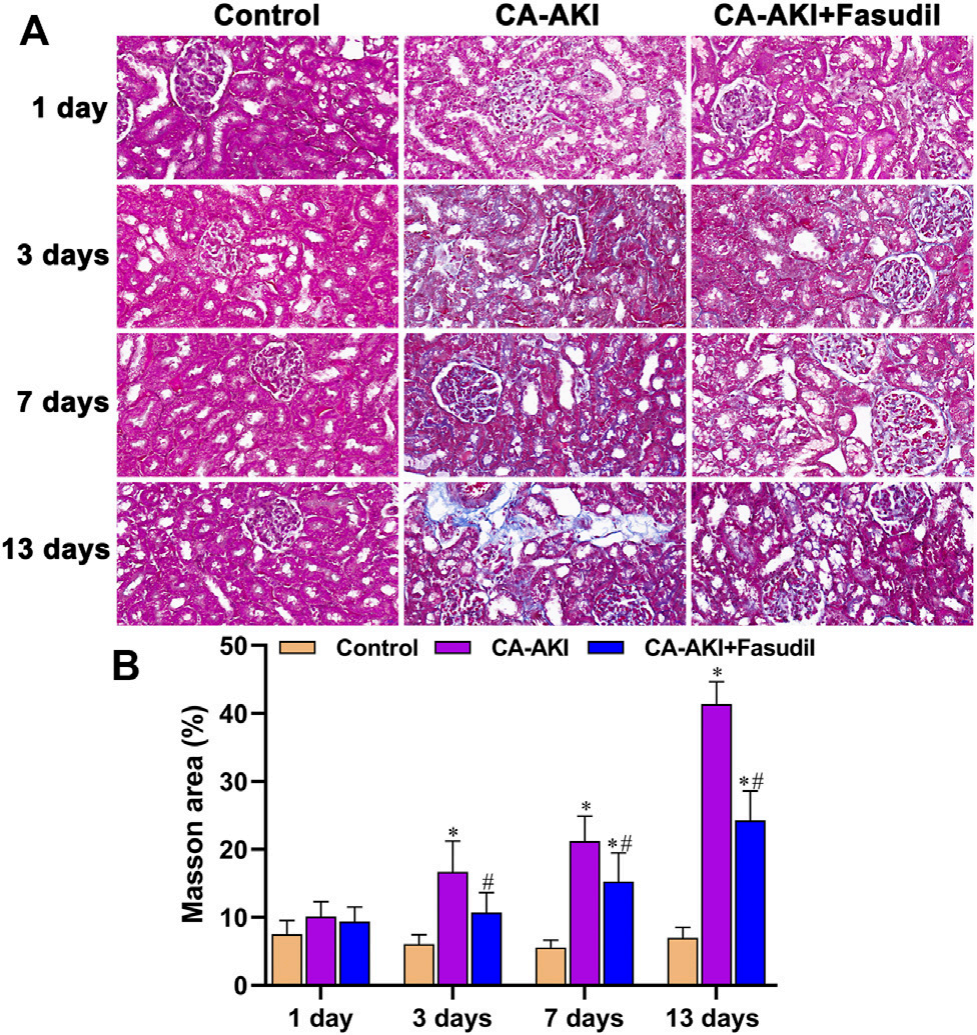
Masson stains of renal fibrosis in three animal groups: **(A)** Appraisal of relative renal fibrosis extent (stained blue); **(B)** Masson’s trichrome staining scores.^*^ *p* < 0.05 between CA-AKI/CA-AKI + Fasudil and controls; ^#^ *p* < 0.05 between CA-AKI + Fasudil and CA-AKI.

Using Masson’s stain, glomerular, renal tubular, and interstitial collagen deposition proved significantly greater in rats with CA-AKI (vs. controls) at most time points (Day 3, *p* < 0.0001; Day 7, *p* < 0.0001; Day 13, *p* < 0.0001). Compared with rats of the CA-AKI group, those of the CA-AKI + Fasudil group showed significantly less collagen deposition in interstitial and glomerular areas on Days 3, 7, and 13 (*p* = 0.018, *p* = 0.020, and *p* < 0.0001, respectively) (Figure 7).

### Effects of Fasudil on Rho Kinase (ROCK) Expression and MYPT1 Phosphorylation

**FIGURE 8:**
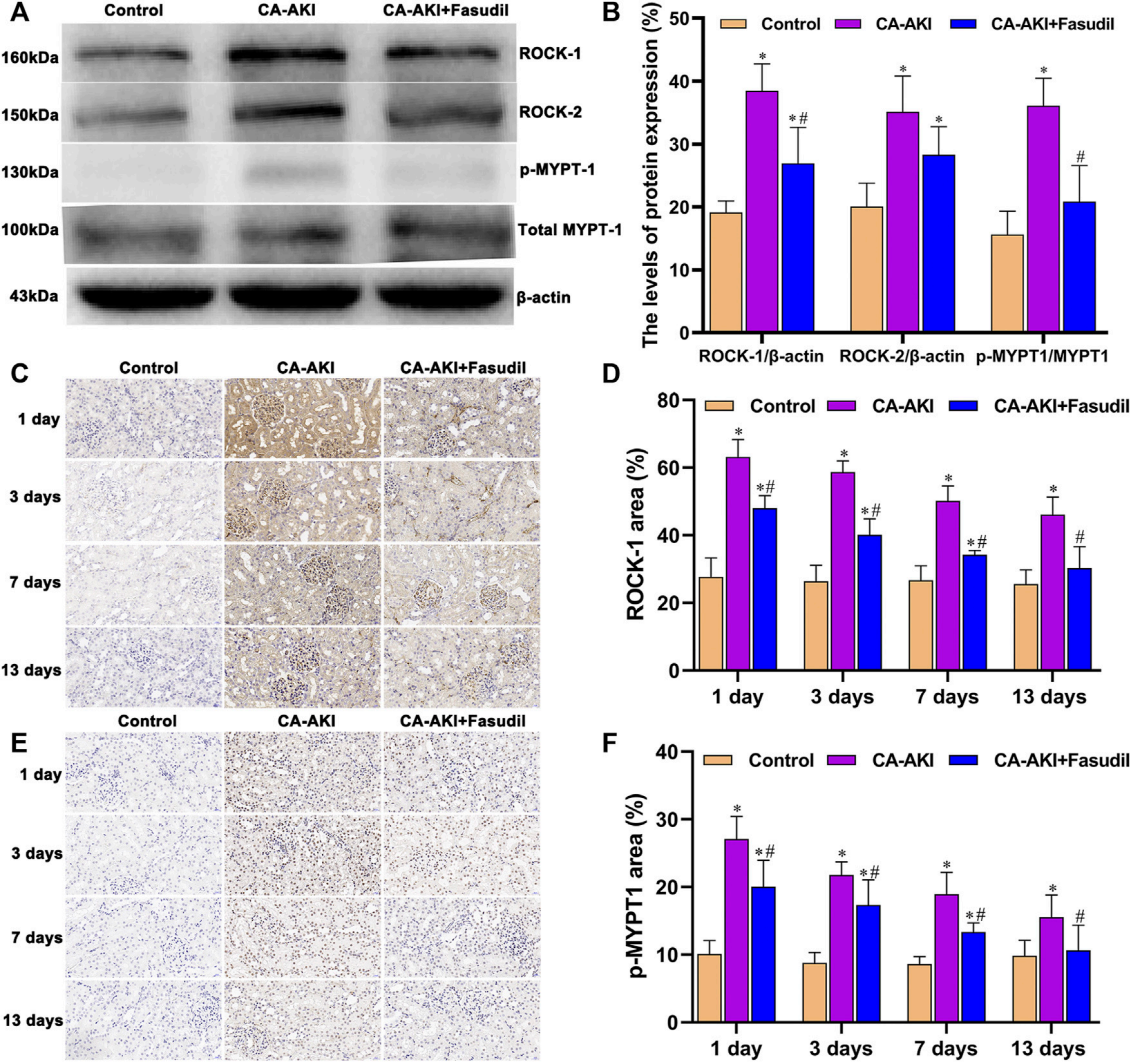
Rho/ROCK expression in three animal groups: **(A)** expression levels of ROCK-1, ROCK-2, MYPT1, and p-MYPT1 by group (western blot); **(B)** relative densitometry analysis of protein ratios; **(C)** ROCK-1 expression by group; **(D)** ROCK-1 scores by group; **(E)** p-MYPT1 expression by group; and **(F)** p-MYPT1 scores by group.^*^ *p* < 0.05 between CA-AKI/CA-AKI + Fasudil and controls; ^#^ *p* < 0.05 between CA-AKI + Fasudil and CA-AKI.

The levels of ROCK proteins (ROCK-1 and ROCK-2) were also measured to examine molecular mechanisms of their inhibition by fasudil. Treatment conferred renal protection on Day 3 in our rat model of CA-AKI. As shown in Figure 8A, fasudil administration significantly reduced the otherwise significant upregulation of ROCK-1 by iopromide injection in untreated rats with CA-AKI (*p* = 0.001). However, no statistical difference was observed in ROCK-2 between the CA-AKI + Fasudil group and the CA-AKI group (*p* = 0.07) (Figure 8B). There was also a progressive decline of ROCK-1 expression in the CA-AKI + Fasudil (vs. CA-AKI) group following iopromide injection (Days 1–13) (Day 1, *p* < 0.0001; Day 3, *p* < 0.0001; Day 7, *p* < 0.0001; Day 13, *p* < 0.0001), as shown by immunostain (Figures 8C,D).

Expression of p-MYPT1 intensified after iopromide injection (reflecting increased ROCK activity) and was similarly attenuated by fasudil. Rats in the CA-AKI group demonstrated a significantly higher p-MYPT1/MYPT1 ratio, compared with the CA-AKI + Fasudil group (*p* < 0.0001) (Figures 8A,B). Immunostaining revealed significantly more accumulation of p-MYPT1 in the CA-AKI (vs. CA-AKI + Fasudil) group at all time points (Day 1, *p* = 0.005; Day 3, *p* = 0.028; Day 7, *p* = 0.001; Day 13, *p* = 0.048) (Figures 8E,F).

### Effects of Fasudil on HIF-1α Expression and PHD2 Activation

**FIGURE 9:**
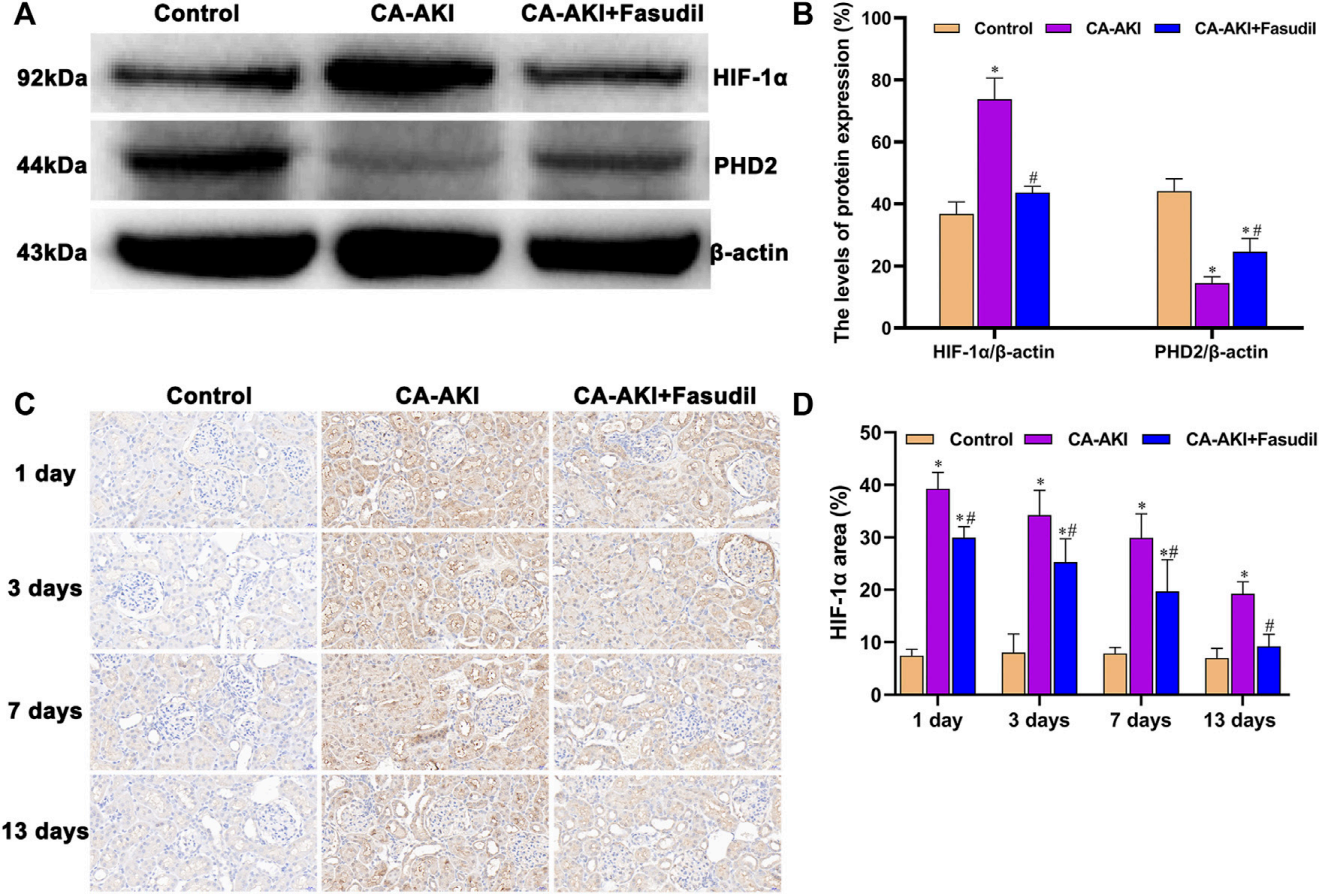
HIF-1α/PHD2 expression in three animal groups: **(A)** expression levels of HIF-1α and PHD2 by group (western blot); **(B)** relative densitometry analysis of protein ratios; **(C)** HIF-1α expression by group; and **(D)** HIF-1α scores by group.^*^ *p* < 0.05 between CA-AKI/CA-AKI + Fasudil and controls; ^#^ *p* < 0.05 between CA-AKI + Fasudil and CA-AKI.

Of note, the *in vivo* data we generated confirmed a rise in HIF-1α expression and a decline in expression of PHD2 for rats with CA-AKI. They also indicated a rise in PHD2 transcription and translation from fasudil use for CA-AKI, with inhibition of HIF-1α expression (Figure 9A). Briefly, significantly lower expression of HIF-1α protein was found in fasudil-treated (vs. untreated) rats with CA-AKI (*p* < 0.0001); and PHD2 protein concentrations were significantly higher in the CA-AKI + Fasudil (vs. CA-AKI) group (*p* = 0.001) (Figure 9B).

On Day 1, a significant increase in nuclear accumulation of HIF-1α was detected in immunostained kidney sections from the CA-AKI group. Thereafter, HIF-1α expression levels declined over time but were still detectable on Day 13 (Figure 9C). Expression of HIF-1α in the CA-AKI + Fasudil (vs. CA-AKI) group was significantly reduced at all time points (Day 1, *p* < 0.0001; Day 3, *p* = 0.008; Day 7, *p* = 0.004; Day 13, *p* < 0.0001) (Figure 9D).

### Effects of Fasudil on Expression Levels of TGF-β1 and Smad3

**FIGURE 10:**
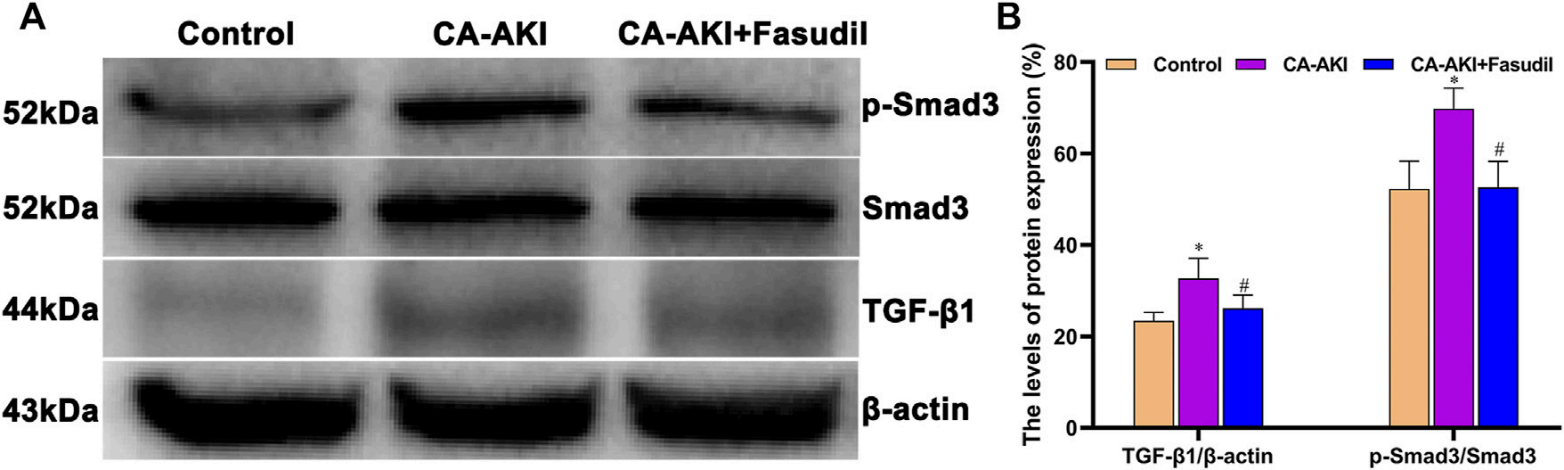
TGF-β1/Smad3 expression in three animal groups: **(A)** expression levels of TGF-β1, Smad3, and p-Smad 3 by group (western blot) and **(B)** relative densitometry analysis of protein ratios.^*^ *p* < 0.05 between CA-AKI/CA-AKI + Fasudil and controls; ^#^ *p* < 0.05 between CA-AKI + Fasudil and CA-AKI.

Three days after iopromide injection, TGF-β1 expression increased significantly in rats with CA-AKI. However, this was significantly attenuated by fasudil administration. Rats in the CA-AKI + Fasudil group demonstrated a significantly lower TGF-β1, compared with the CA-AKI group (*p* = 0.009). Smad3 proteins are known as the main intracellular components of the TGF-β1 signaling pathway. Compared with controls, Smad3 phosphorylation was significantly upregulated in rats with CA-AKI (*p* < 0.0001). Again, this manifestation was reversed by fasudil use. As a result, the ratio of phosphorylated to total Smad3 was significantly lower in fasudil-treated (vs. untreated) rats with CA-AKI (*p* < 0.0001), remaining similar to controls (*p* = 1.000) (Figures 10A,B).

### Effects of Fasudil on Epithelial-Mesenchymal Transition Process

**FIGURE 11:**
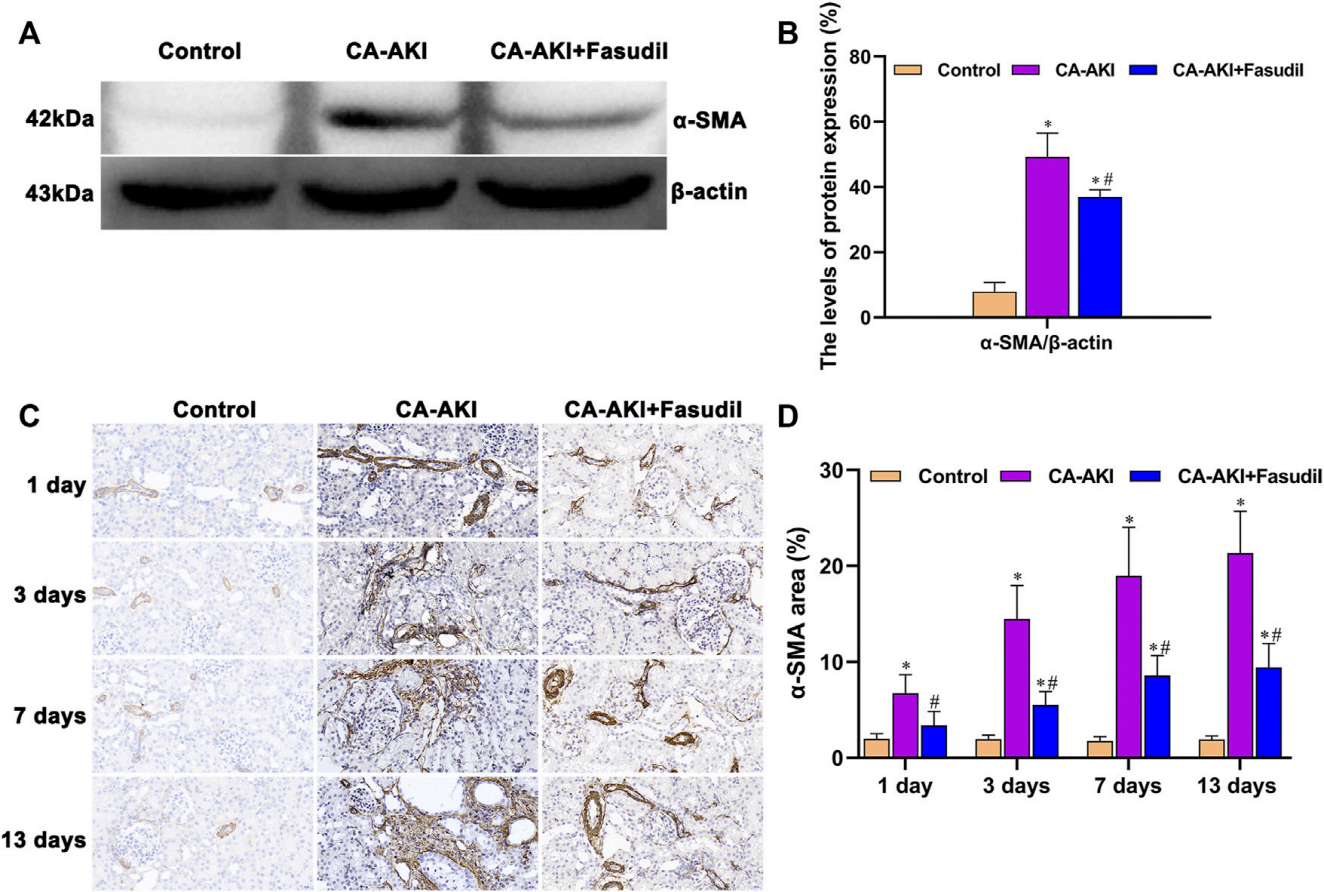
α-SMA expression in three animal groups: **(A)** expression levels of α-SMA by group (western blot); **(B)** relative densitometry analysis of protein ratios; **(C)** α-SMA expression by group; and **(D)** α-SMA scores by group. ^*^ *p* < 0.05 between CA-AKI/CA-AKI + Fasudil and controls; ^#^ *p* < 0.05 between CA-AKI + Fasudil and CA-AKI.

As shown in Figure 11A, α-SMA expression significantly increased in rats with CA-AKI (compared with controls), but was effectively attenuated by fasudil treatment. The expression of α-SMA in the CA-AKI + Fasudil (vs. CA-AKI) group was significantly lower (*p* = 0.023) (Figure 11B). Also, immunostaining showed that α-SMA was primarily found in glomeruli but most prominently in renal tubules of rats with CA-AKI throughout the study (Figure 11C). Compared with controls, α-SMA expression in the CA-AKI group was significantly increased on Days 1 (*p* = 0.004), 3 (*p* = 0.001), 7 (*p* = 0.001), and 13 (*p* < 0.001); yet compared with CA-AKI group, renal α-SMA level in the CA-AKI + Fasudil group was significantly lower on Days 1 (*p* = 0.022), 3 (*p* = 0.002), 7 (*p* = 0.007), and 13 (*p* < 0.0001) (Figure 11D).

### Correlation Between mpMRI and Pathological Scores and HIF-1α Expression

**FIGURE 12:**
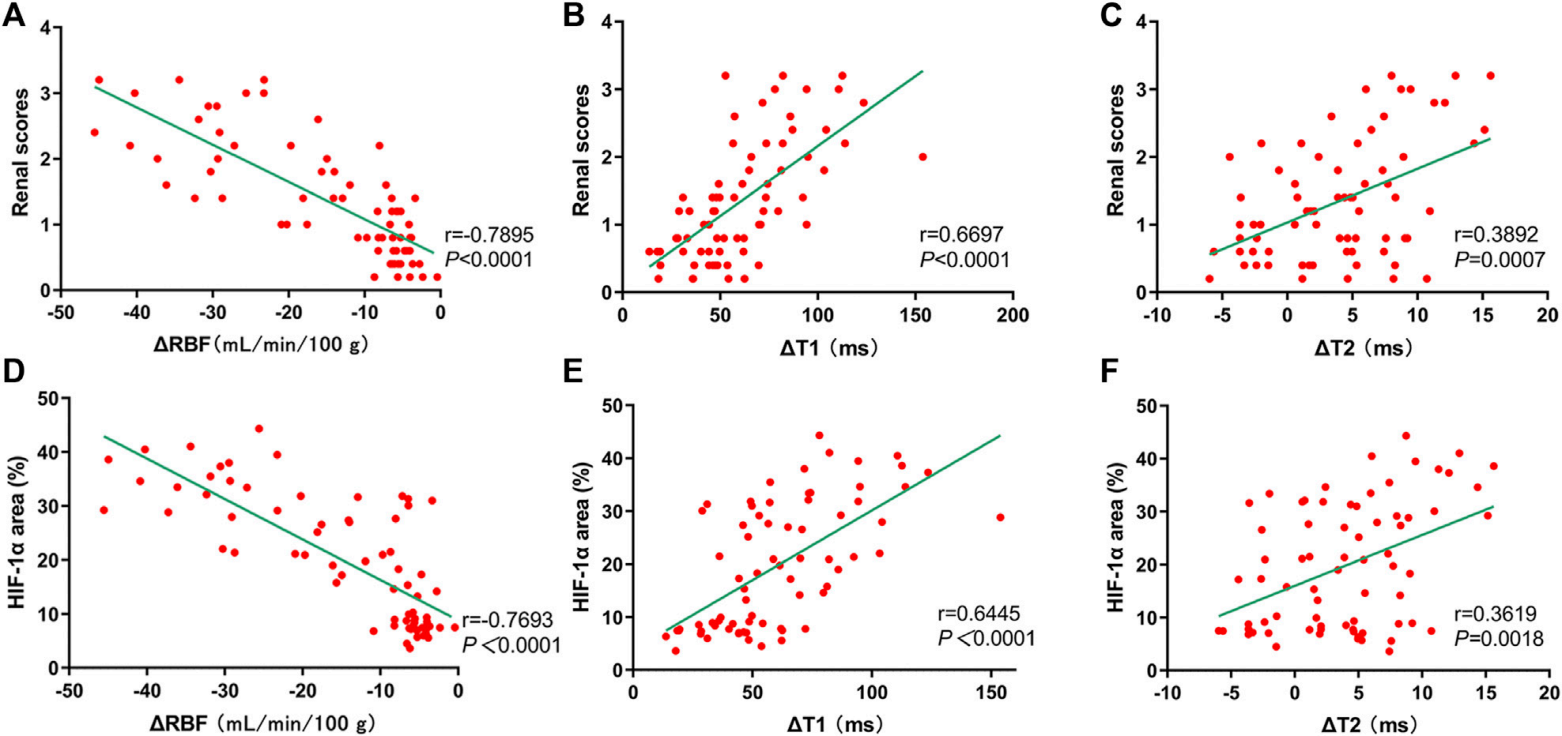
Correlation of mpMRI with renal scores and HIF-1α expression. **(A–C)** mpMRI vs. renal scores: **(A)** ΔRBF values, r = −0.7895; **(B)** ΔT1 values, r = 0.6697; **(C)** ΔT2 values, r = 0.3892; **(D–F)** mpMRI vs. HIF-1α: **(D)** ΔRBF values, r = −0.7693; **(E)** ΔT1 values, r = 0.6445; **(F)** ΔT2 values, r = 0.3619.

Renal injury scores exhibited significantly negative correlation with ΔRBF (r = −0.7895, *p* < 0.0001) (Figure 12A), and significantly positive correlation with ΔT1 values (r = 0.6697, *p* < 0.0001) (Figure 12B), and weakly positive correlation with ΔT2 values (r = 0.3892, *p* = 0.0007) (Figure 12C). In addition, HIF-1α exhibited well correlation with ΔRBF (r = −0.7693, *p* < 0.0001) (Figure 12D), and ΔT1 values (r = 0.6445, *p* < 0.0001) (Figure 12E), while there was weak correlation between HIF-1α and ΔT2 values (r = 0.3619, *p* = 0.0018) (Figure 12F).

## Discussion

In the present study, kidneys of iopromide-treated rats (vs. controls) demonstrated irreversible dysfunction, diminished RBF, inflammatory cell influx, hypoxic injury with tubulointerstitial fibrosis, and structural alterations. However, such changes may be effectively prevented by fasudil inhibition of ROCK. Our data showed that fasudil lowered serum IL-1β levels in the 13 days after CA-AKI. Likewise, ΔT1 and ΔT2 values were significantly decreased in the CA-AKI + Fasudil (vs. CA-AKI) group. It was also apparent that fasudil may increase RBF, combat medullary hypoxia, and limit renal fibrosis through various signaling pathways (Rho/ROCK, HIF-1α, and TGF-β1) and expression of α-SMA. Hence, its promise as a preventative therapy for CA-AKI is mounting.

Our rat model established a multiparametric MRI protocol for the first time for noninvasive quantitation and characterization of renal structural and functional injury in fasudil-treated animals with CA-AKI. This particular protocol enrolling the ASL and T1/T2 mapping enabled the assessment of various pathologic changes at once, including the renal vasculature, perfusion, edema incitement, cellular infiltration, and fibrosis. The therapeutic endpoint of fasudil is improved renal microcirculation, demonstrated by observed RBF increases in the CA-AKI + Fasudil group. We also found that fasudil intervention lowered T1 values at CO, OSOM, and ISOM for the duration of testing (Days 1–13), and lower T2 values at three compartments for the duration of the first 3 days, owing to the alleviation of iopromide-induced tubular detriment, renal parenchymal edema, and infiltration of inflammatory cells. Although the half-life of most CMs is ∼2–7 h, related tissue swelling and tubular damage reportedly last for a relatively long period (Zhang et al., 2012). On the other hand, increased T1 values after CA-AKI can be related to the decrease in renal blood flow, because T1 values depend not only on tissue property but also on kidney blood flow (Dekkers et al., 2020). Hueper et al. also found an increment in the T1 values in ischemia-induced acute kidney injury and in case of hypoperfusion and hypoxemia in OSOM (Hueper et al., 2014). Moreover, the fall in T1 values achieved by fasudil may be partly attributable to inhibition of renal fibrosis, confirmed in Masson’s trichrome and α-SMA immunostain preparations. It has been claimed that in rats with unilateral ureteral obstruction, T1 values correlate significantly with both staining procedures (Hu et al., 2019). A recent report has also demonstrated that T1 mapping may be beneficial in assessing the pathology of renal transplantation, helping to explain histologic changes (cell infiltration, interstitial fibrosis) of acute transplant rejection in mice (Hueper et al., 2016). Besides, our results showed that T1 and T2 tend to be elevated, and RBF degraded, at the second scan in the control rats. The reason may due to the fact that the rats were injected with the sodium pentobarbital twice, which was consistent with the preclinical report (Niles et al., 2015). However, this hypothesis needs to be confirmed by expanding the sample size in future studies.

One should be noted is that the fasudil activity in the context of CA-AKI involves a complex signaling network. The Rho/ROCK inhibition achieved herein may thus reflect a dependent pathway for its renoprotective effect. ROCK is a member of protein kinase A [PKA]/protein kinase G/protein kinase C family of serine/threonine kinases (Wang et al., 2018). We confirm that ROCK-1 protein expression, but not ROCK-2, plays a major role in the regulation of CA-AKI. They may be related to the primary expression of ROCK-1 and ROCK-2 in organs is different. ROCK-1 is more remarkable in kidneys, liver, as well as testis, whereas ROCK-2 is more remarkable in skeletal muscle and brain (Shi and Wei 2007). Consistent with our results, Su et al. have also suggested that ROCK-1 may take over apoptotic signaling in CA-AKI (Su et al., 2014). Moreover, our study has demonstrated that fasudil significantly reduced the levels of phosphorylated MYPT-1. The latter has been recognized by others as means of assessing ROCK activity in the kidney (Su et al., 2014; Seccia et al., 2020), implying that Rho/ROCK pathway inhibition may be useful for moderating or treating CA-AKI.

The basis for CA-AKI is acute hypoxia, a known byproduct of CM use (Heyman et al., 2008). The persistent state of venous stasis that develops after CM injection likely increases cardiac afterload, slowing circulatory blood flow and the rate of renal perfusion (Chen et al., 2015). A recent research has determined that HIF-1α is a crucial regulator of renal hypoxia, particularly in the setting of CA-AKI (Li et al., 2021). Our rat model investigation is aligned with its results, showing that fasudil treatment may significantly reduce HIF-1α expression in areas of glomerular and tubulointerstitial CM-related damage. Fasudil thus exerts a nephroprotective effect, achieved in part by mitigating hypoxia via HIF-1α pathway inhibition.

There are several suggested protective mechanisms of fasudil on the HIF-1α pathway, drawn from our observations and those of others. One explanation is that it reduces the generation of superabundant reactive oxygen species (ROS) that leads to oxidative stress (Wang et al., 2018). It has been found that ROS might directly injure vascular and tubular linings, worsening renal parenchymal hypoxia due to tubular transport dysregulation and endothelial dysfunction (Wang et al., 2019a). Another possibility, supported by functional renal MRI, is that fasudil promotes dilatation in vascular beds and increases renal blood flow. Last, our data indicate that inhibition of HIF-1α signaling through PHD2 activation is pivotal. HIF-1α protein’s degradation is strictly controlled by site-specific hydroxylation, which PHD isoforms mediate (Rajendran et al., 2020). Consequently, ROCK inhibition might facilitate HIF-1α hydroxylation via activation of renal PHD2.

Research increasingly tends to support the view that renal fibrosis in the course of long-term CA-AKI is inflicted by a host of insults, for instance, stress molecules and growth factors (Sharma et al., 2020). The process of renal fibrosis has several crucial phases, including excessive and progressive accumulation of extracellular matrix (ECM), activation of renal interstitial fibroblasts, EMT, and inflammatory cell infiltration (Ferenbach and Bonventre 2015). We were subsequently inclined to investigate whether inhibition of renal fibrosis may be beneficial in ameliorating the long-term damage of CA-AKI.

The present effort has documented that fasudil downregulates TGF-β1 and p-Smad3 expression levels and inhibits α-SMA induction during CA-AKI, which may profoundly reduce renal fibrosis over relatively long periods. This finding is evidenced by the animal model of Park et al., wherein ROCK inhibitors reduced cyclosporine-induced kidney injury by limiting both ECM accumulation and α-SMA expression (Park et al., 2011). Earlier investigations have produced similar findings, i.e., reduced TGF-mRNA expression, suppression of accumulated ECM, and muted α-SMA expression by ROCK inhibitors (Fu et al., 2006; Zhou et al., 2011). It is then reasonable to presume that fasudil may impact CA-AKI pathogenesis long-term, so a focus on renal fibrosis may serve to clarify its underlying mechanism.

In the course experimentation, serum CysC, IL-1β and urinary NGAL levels continued to climb until Day 13, proving that even cursory renal contact with CM is sufficient to provoke long-term injury. More importantly, fasudil inhibited the release of proinflammatory cytokine IL-1β in rats with CA-AKI. As in a prior publication, Wang et al. have shown that fasudil effectively reduces CM-induced inflammatory cell infiltration and cytokine release (Wang et al., 2018). Since fasudil inhibits ROS generation, it may directly reduce the levels of inflammatory cytokines and adhesion molecules directly, and indirectly suppress the inflammatory cell influx (Lu et al., 2013). In addition, the results showed that the changes in mpMRI were much more sensitive than those of CysC and NGAL, which suggests that mpMRI may have a great potential for monitoring the treatment effects of fasudil on CA-AKI.

This study is not without limitations. Although preclinical trial data attest to the efficacy and safety of fasudil in treating CA-AKI at 24 h (Wang et al., 2018), our work brings to light a need for efficacy assessment over a longer observation period (Days 1–13). Since fasudil is a vasodilator, another issue is the lack of a positive control group (only fasudil injection) that could be set to exclude effects on renal blood volume when exploring the effects of fasudil on CA-AKI progression. Finally, we concentrated entirely on renal hypoxia as the perpetrator of CA-AKI. Given the importance of HIF-1α in tubulointerstitial fibrosis (Matoba et al., 2013), a better understanding of cross-talk between ROCK and HIF-1α in tubulointerstitial fibrosis is an important goal for future study.

In conclusion, our study relied on technical advances (T1/T2 mapping and ASL) for new insights into CA-AKI pathogenesis and the early promise of fasudil as a potential treatment. The aim was to identify key timeline events responsible for its therapeutic effects. We have demonstrated that fasudil reduces renal TGF-β1/p-Smad3, HIF-1α/PHD2 signaling activation, and the expression of α-SMA in this setting. Given the importance of Rho/ROCK pathway inhibition in the development of CA-AKI, these revelations could help bolster the attraction of fasudil as a treatment option.

## Data Availability

The raw data supporting the conclusions of this article will be made available by the authors, without undue reservation.
