# Supplementary material for: Assessment of Fasudil on Contrast-Associated Acute Kidney Injury Using Multiparametric Renal MRI
Source: Front Pharmacol. 2022 Jun 15;13:905547. doi: 10.3389/fphar.2022.905547 (PMC9242620; doi:10.3389/fphar.2022.905547)
Supplement: Supplementary file 1 [file DataSheet1.zip › 10.3_supplementary material/Supplemental table1_baselines.docx]

**Table 1. Overview of 13-day multiparametric MRI baseline values in kidneys for three animal groups.**

|  |  | **1 day (n=6)** | **3 days (n=6)** | **7 days (n=6)** | **13days (n=6)** |
| --- | --- | --- | --- | --- | --- |
| **Baseline T1 values (ms)** | | |  |  |  |
| **CO** | Control | 969.512±27.273 | 1011.709±36.819 | 980.979±55.730 | 998.896±36.314 |
|  | CA-AKI | 1132.942±57.326 | 1198.761±55.636 | 976.513±36.678 | 969.932±25.359 |
|  | CA-AKI+Fasudil | 1075.417±48.716 | 1132.341±28.470 | 991.747±40.858 | 996.289±21.562 |
| **OSOM** | Control | 829.171±83.339 | 835.364±51.383 | 827.906±50.165 | 832.412±25.209 |
|  | CA-AKI | 922.849±32.676 | 977.496±45.062 | 877.329±21.066 | 839.591±20.284 |
|  | CA-AKI+Fasudil | 880.237±30.076 | 903.090±42.893 | 865.770±56.733 | 851.876±48.861 |
| **ISOM** | Control | 1324.810±49.881 | 1304.842±30.974 | 1294.089±43.422 | 1287.414±36.614 |
|  | CA-AKI | 1325.360±86.856 | 1310.613±39.873 | 1302.693±57.156 | 1307.383±45.759 |
|  | CA-AKI+Fasudil | 1350.618±54.962 | 1348.223±47.567 | 1349.659±55.701 | 1329.781±40.022 |
| **Baseline T2 values (ms)** | | |  |  |  |
| **CO** | Control | 46.170±4.414 | 44.185±3.890 | 45.698±2.057 | 45.676±5.821 |
|  | CA-AKI | 48.160±3.395 | 46.255±3.896 | 48.059±3.252 | 47.004±4.344 |
|  | CA-AKI+Fasudil | 51.588±2.386 | 49.909±3.032 | 48.791±4.059 | 46.599±3.359 |
| **OSOM** | Control | 42.472±4.162 | 40.472±5.208 | 41.985±1.325 | 40.208±1.004 |
|  | CA-AKI | 42.254±8.140 | 41.311±2.763 | 44.031±2.430 | 40.416±3.232 |
|  | CA-AKI+Fasudil | 41.143±1.291 | 42.902±3.584 | 42.920±5.262 | 41.273±2.532 |
| **ISOM** | Control | 53.131±4.810 | 51.434±1.589 | 52.324±3.843 | 53.565±3.260 |
|  | CA-AKI | 53.780±3.058 | 52.115±1.184 | 54.962±2.917 | 54.682±2.560 |
|  | CA-AKI+Fasudil | 55.125±3.090 | 52.828±2.963 | 55.217±3.079 | 54.402±1.124 |
| **Baseline RBF values (mL/min/100g)** | | |  |  |  |
| **CO** | Control | 197.827±9.840 | 188.876±11.882 | 192.174±13.653 | 187.714±9.393 |
|  | CA-AKI | 194.008±16.654 | 191.224±12.470 | 184.955±21.793 | 175.763±9.326 |
|  | CA-AKI+Fasudil | 196.960±9.979 | 183.180±7.955 | 182.221±10.294 | 178.253±9.793 |
| **OSOM** | Control | 166.965±10.134 | 165.115±20.516 | 168.774±15.873 | 162.239±14.430 |
|  | CA-AKI | 161.896±9.770 | 163.777±9.836 | 162.628±21.743 | 146.475±13.258 |
|  | CA-AKI+Fasudil | 167.105±4.748 | 155.922±18.884 | 157.119±22.837 | 159.683±8.265 |
| **ISOM** | Control | 151.972±17.641 | 152.769±18.510 | 149.156±14.570 | 150.899±18.252 |
|  | CA-AKI | 143.866±9.630 | 143.653±8.526 | 143.233±30.264 | 137.559±12.637 |
|  | CA-AKI+Fasudil | 151.697±4.932 | 144.020±12.507 | 137.896±16.963 | 144.321±7.400 |

Mean±SD of T1 relaxation times in cortex (CO), outer stripe of the outer medulla (OSOM) and inner stripe of the outer medulla (ISOM) at different time points in three animal groups.

**Table 3. Laboratory data for three animal groups**

|  |  | 1 day | 3 days | 7 days | 13 days |
| --- | --- | --- | --- | --- | --- |
| **CysC (ng/ml)** | Control | 407.87±37.67 | 412.88±50.08 | 413.58±36.76 | 408.10±35.98 |
|  | CA-AKI | 609.96±27.82^*^ | 581.86±36.18^*^ | 552.63±31.36^*^ | 482.21±28.25^*^ |
|  | Fasudil+CA-AKI | 568.03±33.29^*#^ | 528.91±33.31^*#^ | 509.31±27.94^*#^ | 438.97±26.10^#^ |
| **NGAL (ng/ml)** | Control | 17.82±2.48 | 18.18±2.87 | 17.57±2.16 | 17.12±1.87 |
|  | CA-AKI | 31.81±3.02^*^ | 28.84±2.84^*^ | 26.92±2.32^*^ | 23.65±2.85^*^ |
|  | Fasudil+CA-AKI | 27.78±2.85^*#^ | 24.77±2.91^*#^ | 23.43±2.49^*#^ | 19.84±2.03^#^ |
| **IL-1β (ng/ml)** | Control | 55.88±5.92 | 52.17±6.78 | 55.26±3.91 | 52.20±4.94 |
|  | CA-AKI | 126.55±6.23^*^ | 116.71±6.11^*^ | 91.12±3.79^*^ | 81.12±13.17^*^ |
|  | Fasudil+CA-AKI | 95.65±6.93^*#^ | 84.16±7.30^*#^ | 64.13±5.03^*#^ | 57.85±3.58^#^ |

^*^*p*<0.05 vs controls, ^#^*p*<0.05 vs CA-AKI group
